# A General Approach for the Modelling of Negative Feedback Physiological Control Systems

**DOI:** 10.3390/bioengineering10070835

**Published:** 2023-07-14

**Authors:** Alfonso Maria Ponsiglione, Francesco Montefusco, Leandro Donisi, Annarita Tedesco, Carlo Cosentino, Alessio Merola, Maria Romano, Francesco Amato

**Affiliations:** 1 Dipartimento di Ingegneria Elettrica e delle Tecnologie dell’Informazione, Università degli Studi di Napoli Federico II, Via Claudio 21, 80125 Napoli, Italy; 2 Dipartimento di Scienze Economiche, Giuridiche, Informatiche e Motorie, Università degli Studi di Napoli Parthenope, 80035 Nola, Italy; 3 Dipartimento di Scienze Mediche e Chirurgiche Avanzate, Università degli studi della Campania “Luigi Vanvitelli”, P.zza L. Miraglia 2, 80138 Napoli, Italy; leandro.donisi@unicampania.it; 4 Dipartimento di Ingegneria per l’Innovazione, Universitá del Salento, 73100 Lecce, Italy; 5 School of Computer and Biomedical Engineering, Dipartimento di Medicina Sperimentale e Clinica, Università degli Studi Magna Græcia di Catanzaro, Campus di Germaneto “Salvatore Venuta”, 88100 Catanzaro, Italy

**Keywords:** physiological systems, negative feedback control systems, stability, homeostasis

## Abstract

Mathematical models can improve the understanding of physiological systems behaviour, which is a fundamental topic in the bioengineering field. Having a reliable model enables researchers to carry out in silico experiments, which require less time and resources compared to their in vivo and in vitro counterparts. This work’s objective is to capture the characteristics that a nonlinear dynamical mathematical model should exhibit, in order to describe physiological control systems at different scales. The similarities among various negative feedback physiological systems have been investigated and a unique general framework to describe them has been proposed. Within such a framework, both the existence and stability of equilibrium points are investigated. The model here introduced is based on a closed-loop topology, on which the homeostatic process is based. Finally, to validate the model, three paradigmatic examples of physiological control systems are illustrated and discussed: the ultrasensitivity mechanism for achieving homeostasis in biomolecular circuits, the blood glucose regulation, and the neuromuscular reflex arc (also referred to as muscle stretch reflex). The results show that, by a suitable choice of the modelling functions, the dynamic evolution of the systems under study can be described through the proposed general nonlinear model. Furthermore, the analysis of the equilibrium points and dynamics of the above-mentioned systems are consistent with the literature.

## 1. Introduction

Mathematical models proved to be a powerful and meaningful tool to achieve a comprehensive understanding of the behaviour and dynamics of physiological systems, which is a fundamental topic in the bioengineering field; see, for instance, the books [1,2,3,4] that, still today, represent a cornerstone for the researchers of the field. Looking to more recent times, one can refer to the monographs [5,6,7]. Indeed, having a reliable and accurate physiological model enables researchers to carry out in silico experiments, which require far less time and resources compared to their in vivo and in vitro counterparts.

Most of the physiological systems, taken at their own different scales, prove to have analogous and overlapping control dynamics. It is possible to consider, as examples, at the nano-scale, the genetic circuits for biomolecular process control, described in [8,9,10,11,12,13,14] and in the relevant books [15,16,17] with their bibliographies, and at the micro- and milli-scale, as the cell population dynamics involved, for instance, in the generation and growth of tumours [18,19,20,21,22] and the regulation of sleep–wake cycles [23]. Finally, examples at the macro-scale level are the control systems for the regulation of cardiovascular processes, neuromuscular activities, respiration dynamics, etc. [7,24,25,26,27,28,29,30,31]. This category of systems incorporates a negative feedback control scheme, within a closed-loop configuration, which permits the values of the physiological parameters to be kept near to the equilibrium itself in order to preserve the homeostatic equilibrium [7,32].

Even if a physiological control system [7] and a conventional (artificial) closed-loop negative feedback system [33] have fundamentally the same objective, i.e., reaching and maintaining a reference value (at least, not to be far from it), their functioning and implementation is rather different. In the former, the controller and the plant, whose output must closely match the reference signal, are separated physically and logically. The role of the controller consists of developing and implementing the rules to control the system based on the tracking error measurement. In the latter, it is unnecessary to specify the error signal because an “external” reference signal is absent; indeed, as further explained in the following, the physiological system itself acts for reaching the reference status, which is intrinsically embedded in the system. Furthermore, the topology of physiological systems is peculiar and different from the artificial ones; indeed, there is neither conceptual nor systemic difference between the dynamics to be controlled (i.e., what is classically defined to be the plant) and the driving dynamics (i.e., what is considered to be the controller), because the two components are, at the same time, the cause and effect of the behaviour of the other.

The aim of this research work is to introduce a general framework to represent and investigate the behaviour and dynamics of negative feedback physiological control systems characterised by the presence of two species interacting with each other. In this regard, it is worth recalling that the model of a system must not be confused with the system itself; indeed, for the same physical process, it is possible to develop different models as the complexity of the dynamics that are taken into account increases. The complexity depends on both the order of the model, i.e., the number of the state variables that are involved, and the kind of relationships, i.e., the degree of linearity, between the state variables themselves.

Typically, the mathematical modeller reaches a compromise between the complexity of the model and the adherence to the real system, because the computational burden grows with that of the order of the model that has been developed. When dealing with negative feedback systems, the description through a low-order model is encouraged by the fact that a well-designed closed-loop system can be usually described, in first approximation, by a first- or second-order linear model [33].

In this framework, the objective of this work is to develop a rather general and simple model structure able to capture the fundamental dynamics on which most physiological systems are based. In particular, the homeostasis is here considered. Let us briefly recall that homeostasis is a physiological process aimed at maintaining the steady state, or, more exactly, the vital parameters of human cells, organs, or systems, within a set range by means of self-regulatory mechanisms [34]; hence, basically, it is a negative feedback system. The proposed model is composed of a set of two nonlinear ordinary differential equations (ODEs), where each term of the equations has a well-defined function in the feedback mechanism underlying the physiological process and a precise mathematical characterisation. It is well-known that many papers, dealing with the modelling of physiological systems, have been published in the literature [32,35,36,37,38,39,40]. However, they have some drawbacks, such as, for example, to investigate only the anatomical issues; hence, they can only describe specific cases. Against that, the model here proposed has the advantage of being very simple and general, about the homeostatic process, but, simultaneously, able to be adaptable to specific cases by choosing in a suitable way the modelling functions.

In order to validate the proposed approach and to illustrate its generality from the nano- up to the macro-scale, three paradigmatic examples are discussed in detail: the first one concerns the role of ultrasensitivity in biomolecular circuitry for achieving homeostasis; the second one regards the mechanism which regulates the glucose concentration in the plasma; and the third one regards the neuromuscular reflex arc. The examples illustrated in this paper will demonstrate that each of them falls within the general model or, vice versa, that the proposed model, despite its simplicity, is actually able to capture the dynamic evolution of homeostatic physiological processes.

The second part of this paper is devoted to studying the properties of the equilibrium points that the proposed general model can exhibit. This investigation is, in some way, the ultimate goal of the research because, as said before, concerning the dynamics of a given negative feedback physiological process, the concept of the equilibrium point is strictly related to the maintenance of the homeostatic balance for that process (see, e.g., [32,38] and the bibliography therein), which represents a fundamental mechanism of the physiology of any living being.

To this end, a sufficient condition that guarantees the existence of at least one point of equilibrium for the proposed general model, and its exponential stability, is provided. In some cases, which cover some situations of practical interest, it will be proved that an equilibrium point, if existing, is automatically guaranteed to be unique and exponentially stable. Such theory is then applied to the above-mentioned case studies to illustrate the effectiveness of the proposed approach.

This paper largely extends the very preliminary study presented in [41], by adding two further examples that, together with the one presented in [41], are discussed in much more detail; also, further theoretical results and investigations regarding the dynamical behaviour around the homeostatic equilibria are provided.

## 2. Methods

As mentioned in the Introduction, to have useful cooperation between some specialised organs of the body, their functions must be continuously adjusted, in order to meet specific needs or maintain a specific range of vital parameters. In other words, they have to be subject to control and regulation; the physiological way to achieve control over the system dynamics is to exploit a negative feedback control configuration, which is also able to respond to unexpected or external disturbances. A physiological control system can operate following two different patterns, that is, either remaining within the organ itself or via a superordinate organ, such as the central nervous system or the endocrine system. However, from a conceptual point of view, the control circuit is the same [34].

### 2.1. Reference Model for a Physiological Control System

**Figure 1:**
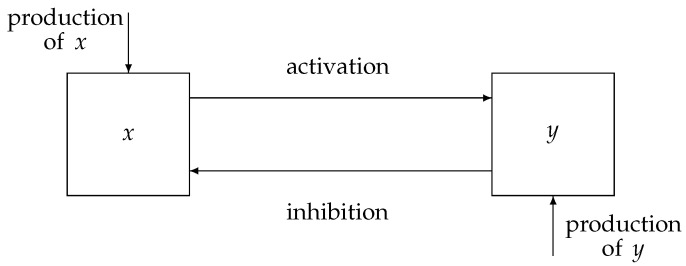
Schematic representation of a negative feedback physiological control system. Adapted with permission from [41].

A representation of a physiological system with a negative feedback control loop is displayed in Figure 1, where the state variables of the proposed model, *x* and *y*, indicate the activation and inhibition species, respectively. Indeed, the interplay between the state variables of the system is based on an activation mechanism of *x* on *y* that causes *y* to increase and, as a consequence, enables the inhibition mechanism of *y* on *x*, which thereby decreases. Therefore, typical dynamics of a negative feedback control system are implemented, where the augmentation of *y* (effect) reduces the accumulation of *x* (cause). Note that, in comparison with traditional negative feedback systems, explicit input/output signals are missing. Indeed, in many physiological systems, *x* and *y* can change their role, being, alternatively, the input/output of the system.

A convenient description to capture such a physiological mechanism, which in turn is dependent on the species interactions, is given by

(1a) $$\begin{array}{cc} \dot{y}\left(t\right) & =-\alpha y\left(t\right)+u_{y}\left(t\right)+f\left(x(t),y(t)\right) \end{array}$$

(1b) $$\begin{array}{cc} \dot{x}\left(t\right) & =-\beta x\left(t\right)+u_{x}\left(t\right)+g\left(x(t),y(t)\right)\;. \end{array}$$

On one hand, $u_{y}$ represents the production rate of the *y* species, while the activation function f(·,·) describes the mechanism of activation of *x* against *y*. On the other hand, $u_{x}$ indicates the rate of production of the *x* species, while the inhibition function g(·,·) accounts for the mechanism of inhibition of *y* against *x*. As for α and β, defined as positive scalars, they are those model’s parameters representing the consumption rates of *y* and *x*, respectively.

The following assumptions on the functions involved in the ODEs (1) are made.

**Assumption** **1.** *Concerning model *(1)*, the following hold.*

(*i*) *Both the activation and the inhibition functions, i.e., f(·,·) and g(·,·), respectively, are piecewise continuously differentiable mappings from $R^{+}\times R^{+}$ to R.*
(*ii*) *For any given $y\in R^{+}$, the activation function f(·,y) is non-decreasing and the inhibition function g(·,y) is non-increasing.*
(*iii*) *For any given $x\in R^{+}$, the activation function f(x,·) is non-decreasing and the inhibition function g(x,·) is non-increasing.*
(*iv*) *The production rates are assumed both endogenous and constant in time, i.e., $u_{x}\left(t\right)=u_{x}\in R^{+}$, and $u_{y}\left(t\right)=u_{y}\in R^{+}$.*

Some comments are in order to clarify the rationale underlying Assumption 1.

**Remark** **1.** *Assumption 1-(i) guarantees the existence and uniqueness of the solution of model *(1)* starting from any $\left(x_{0},y_{0}\right)\in R^{+}\times R^{+}$, at time t=0, see [42], pp. 470–471. Moreover, Assumptions 1-(ii) and 1-(iii) capture the negative feedback nature of model *(1)*, as will be clarified in the following sections.*

**Remark** **2.** *In general, the production rates $u_{x}$ and $u_{y}$ may both have an endogenous (internal to the organism) and/or an exogenous (external to the organism, for instance, artificial) nature. In this paper, it is assumed that the production is only endogenous; in this case, according to Assumption 1-(iv), it is assumed to be constant, in order to reach the homeostatic equilibrium of the involved physiological system.*

**Remark** **3.** *The possibility of external access to $u_{x}$ and/or $u_{y}$ is of great importance when the problem of artificially controlling a physiological system is faced [43,44], which is not a goal of this paper and will be discussed in future work; in those cases, the external contribution to the production rates can render $u_{x}$ and $u_{y}$ varying in time.*

### 2.2. Comparison between Physiological and Classical Feedback Control Systems

**Figure 2:**
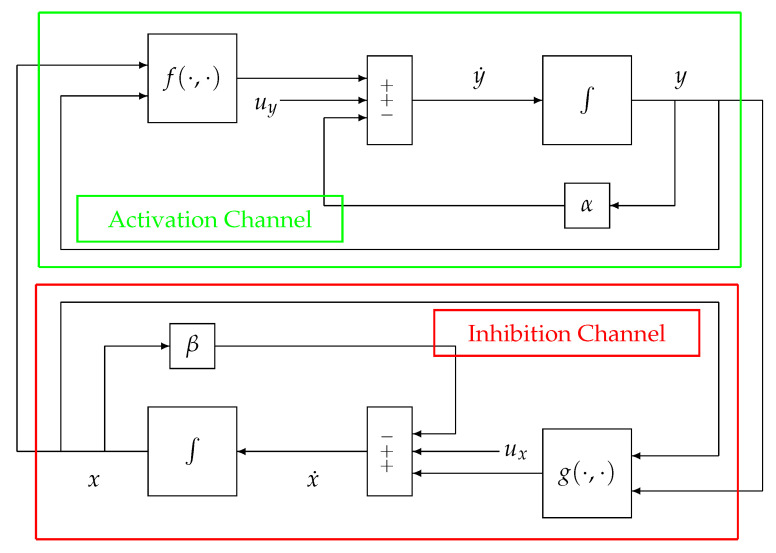
Block components of model (1). Reproduced with permission from [41].

**Figure 3:**
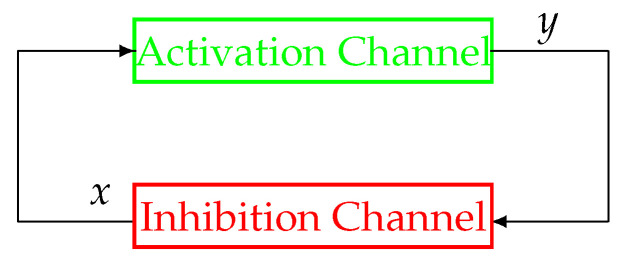
The compact version of the feedback control scheme in Figure 2. Reproduced with permission from [41].

In the following, the block-scheme implementation in (1), represented in Figure 2, will be considered. The scheme can be separated into two parts, each one mirrored by the other, namely, the upper (green) path of the loop, which is referred to as the Activation Channel, and the lower (red) path named the Inhibition Channel. Such a scheme can be represented in an equivalent and more compact way as in Figure 3.

**Figure 4:**
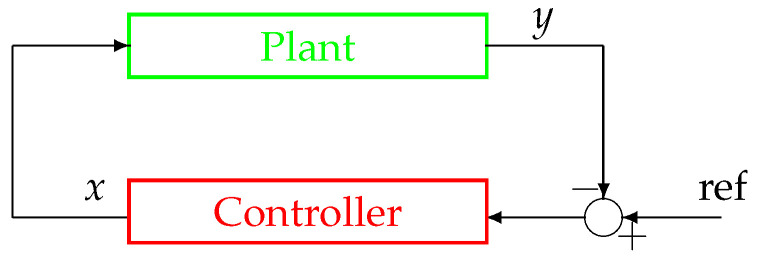
Configuration of a classical negative feedback control system in a closed-loop topology.

In order to present and discuss the peculiar characteristics of the system in Figure 2 and Figure 3, compared to more traditional non-physiological control systems, please refer to Figure 4, which displays the configuration of a classical negative feedback control system in a compact representation.

The first difference between the schemes in Figure 2 and Figure 3 and the scheme in Figure 4 is represented by the node of summation, a fundamental controller’s component in classical systems’ configuration as in Figure 4. It can be observed that this node is missing in physiological systems and, as a consequence, it is impossible to define an error signal (represented by the summation node’s output) in the framework of a physiological system as displayed in Figure 2 and Figure 3. Moreover, similarly to the error signal, the configuration of a physiological system also lacks the reference signal (Figure 4), which, as mentioned in the Introduction, is intrinsically knownto the system. For this reason, those control systems’ configurations such as the one in Figure 2 and Figure 3 are sometimes referred to as a *control system without error detection* [45]. The reference signal is typically a trajectory that the output *y* must follow, while the negative feedback configuration in the closed-loop topology guarantees tracking robustness in spite of perturbations and disturbances acting on the plant.

The second difference can be better explained by looking at Figure 2. The Activation and Inhibition channels display a mirror topology, in which each channel reacts in a way to oppose the variations caused by the other. In this context, it does not make sense to look for a “controller” and a “plant”, although in some cases, in the literature, the inhibition action can be identified with a control action (for example, when *g* models the activity of the nervous system). In other words, there are no conceptual differences between the two channels (i.e., the upper and the lower parts) of the system displayed in Figure 2 and Figure 3. As a consequence of this specular configuration, the control action results to be *embedded* within the model itself and works to direct the whole system towards the achievement of the homeostatic equilibrium.

Conversely, in a classical control system (see Figure 4), the controller, aimed at establishing control laws based on tracking error measurements, is both physically and logically separated from the plant, which is aimed at producing an output that resembles the reference signal; moreover, the controller structure is generally completely different from the one of the controlled process.

In the following, the system in (1) is investigated to highlight the peculiar behaviour of the proposed configuration and to study the conditions for the existence of a feasible homeostatic equilibrium point. Three building physiological examples will be presented to illustrate the most relevant aspects of the work.

### 2.3. A First Paradigmatic Example: Ultrasensitivity for Achieving Homeostasis in Biomolecular Circuitry

In [46], the role of ultrasensitivity, a common nonlinear characteristic of cellular systems for explaining the adaptive response dynamics observed in the yeast osmoregulatory response network, is investigated.

Ultrasensitivity describes a particular form of sensitivity in a biological system, in which the gain of the system changes, in a narrow range, from very low to very high and then back to very low as the magnitude of the input signal increases; in other words, the system does not respond to incoming signals outside of a certain regime but responds in a highly sensitive manner within that regime.

The ultrasensitivity mechanism can be described by the couple of ODEs,

(2a) $$\begin{array}{cc} \dot{y}\left(t\right) & =-\alpha y\left(t\right)+u_{y}+f\left(x(t)\right) \end{array}$$

(2b) $$\begin{array}{cc} \dot{x}\left(t\right) & =-\beta x\left(t\right)+u_{x}+g\left(y(t)\right)\;, \end{array}$$

where *x* and *y* denote the concentrations of the interacting species, $u_{x}$ and $u_{y}$ are constant input fluxes, α and β represent the degradation rates of *x* and *y*, respectively.

The variable *x* activates *y* by the function f(x), whereas *y* inhibits *x* by the function g(y). Note that, in this particular case, *f* depends only on the species *x*, and *g* only on the species *y*.

Because, as said, the model under consideration covers the behaviour of a class of cellular systems dynamic responses, to maintain the discussion at a general level, according to [45,46], a dimensionless normalised value for the parameters is assumed, that is, the following condition is set α=β=1, and the following dimensionless normalised Hill-type expressions [47] for *f* and *g*, with x≥0, are considered

(3a) $$\begin{array}{cc} f(x) & =\frac{x^{m_{x}}}{k_{x}^{m_{x}}+x^{m_{x}}} \end{array}$$

(3b) $$\begin{array}{cc} g(y) & =\frac{k_{y}^{m_{y}}}{k_{y}^{m_{y}}+y^{m_{y}}}\;. \end{array}$$

Note that, independently of the parameter settings, it is guaranteed that f(0)=0, f(∞)=1, g(0)=1, and g(∞)=0.

According to [45,46], the parameters are set as $m_{x}=m_{y}=4$, $k_{x}=0.6$, $k_{y}=0.4$.

**Figure 5:**
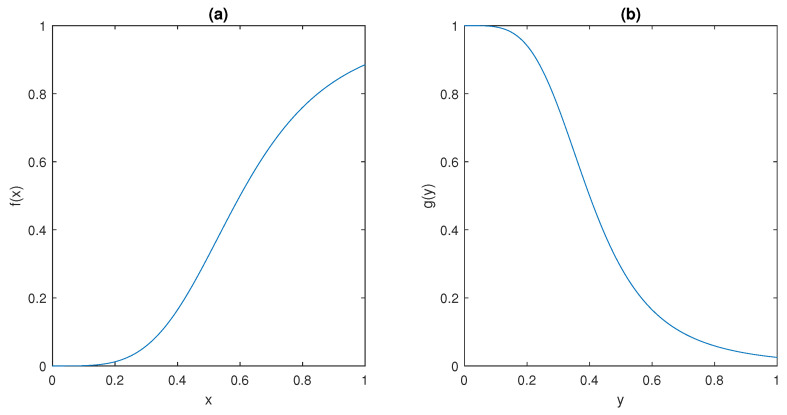
The behaviour of (**a**) *f* and (**b**) *g* in Section 2.3.

The ultrasensitive behaviour of the model is captured by the sigmoidal shape of the functions *f* and *g* depicted in Figure 5; such peculiar behaviour has been observed in many different molecular mechanisms, including dimerisation of transcription factors [48], use of scaffolding proteins in Mitogen-Activated Protein Kinases (MAPK) systems [49], and branching in bacterial phosphorylation/dephosphorylation cycles [50].

It can be observed that f(·) and g(·) plots in Figure 5 show that they satisfy conditions (ii) and (iii) in Assumption 1, respectively.

### 2.4. A Second Paradigmatic Example: Regulation of Blood Glucose Concentration

In this case, a classical negative feedback physiological control system is examined, i.e., blood glucose regulation system, where the main actors are the concentrations of glucose and insulin into the plasma.

According to the review paper [32], a quite general equation to model the activation dynamics is

(4) $$\dot{y}\left(t\right)=-\alpha y\left(t\right)+\Phi_{y}\left(x\left(t\right)\right)\;,$$

where *y* and *x* denote the levels of insulin and glucose, respectively. Insulin is secreted by the pancreatic β-cells at a rate $\Phi_{y}$, which depends on *x*, while it is cleared from the plasma at a rate α.

As said in the Introduction, the level of complexity of the model depends on the class of functions used to model the flux $\Phi_{y}\left(\cdot \right)$. Here, according to [7,51,52], a linear behaviour for $\Phi_{y}$ has been considered, together with a threshold, to take into account the fact that the insulin production ceases when the glucose concentration falls below a given limit, say ϕ; therefore, Equation (4) becomes

(5a) $$\begin{array}{cc} \dot{y}\left(t\right) & =-\alpha y(t)\;,\;0\leq x(t)\leq \phi \end{array}$$

(5b) $$\begin{array}{cc} \dot{y}\left(t\right) & =-\alpha y\left(t\right)+\gamma \left(x(t)-\phi \right)\;,\;x\left(t\right)>\phi \;. \end{array}$$

Equations (5a) and (5b) can be compacted as follows:

(6) $$\dot{y}\left(t\right)=-\alpha y\left(t\right)+f\left(x\right)\;,$$

where

(7) $$f\left(x\right):=\left\{\begin{array}{cc} 0 & 0\leq x\leq \phi \\ \gamma (x-\phi ) & x>\phi \;. \end{array}\right.$$

Note that, in this case, *f* is dependent only on the species *x*, and it is readily seen to satisfy point (ii) in Assumption 1, because γ is a positive scalar.

Analogously, the behaviour of the inhibition mechanism, in accordance with [32], is described by

(8) $$\dot{x}\left(t\right)=u_{x}-\Psi_{x}\left(x(t),y(t)\right)\;,$$

where the term $u_{x}$ represents the constant rate of blood glucose production. The flux $\Psi_{x}\left(\cdot ,\cdot \right)$ accounts for three different ways in which glucose is cleared out; therefore,

(9) $$\Psi_{x}\left(x,y\right)=\Phi_{1}\left(x\right)+\Phi_{2}\left(x,y\right)+\Phi_{3}\left(x\right)\;,$$

where $\Phi_{1}$ is related to the insulin-independent tissue utilisation rate, $\Phi_{2}$ accounts for the insulin-dependent tissue utilisation rate [53], and $\Phi_{3}$ is due to the renal excretion of glucose.

As in the case of the activation Equation (4), even for Equation (9), in order to obtain a simpler model, it is possible to follow [7,52], where $\Phi_{1}$ has a linear structure, $\Phi_{2}$ is a bilinear function, and $\Phi_{3}$ contains a linear term with a threshold; therefore,

(10a) $$\begin{array}{cc} \Phi_{1}\left(x\right) & =-\beta x \end{array}$$

(10b) $$\begin{array}{cc} \Phi_{2}\left(x,y\right) & =-\nu xy \end{array}$$

(10c) $$\begin{array}{cc} \Phi_{3}\left(x\right) & =\left\{\begin{array}{cc} 0 & 0\leq x\leq \theta \\ -\mu (x-\theta ) & x>\theta \;, \end{array}\right. \end{array}$$

where β represents the insulin-independent rate of glucose consumption by the tissues, ν represents the insulin-dependent rate of glucose consumption by the tissues, μ represents the excretion rate of blood glucose, and θ indicates the physiological threshold for the renal discharge of glucose.

Hence, Equation (8) can be rewritten as

(11) $$\dot{x}\left(t\right)=u_{x}-\beta x\left(t\right)+g\left(x,y\right)\;,$$

where

(12) $$g\left(x,y\right):=\left\{\begin{array}{cc} -\nu xy & 0\leq x\leq \theta \\ -\mu (x-\theta )-\nu xy & x>\theta \;. \end{array}\right.$$

In this case, by deriving the function *g* with respect to *x*, Equation (13) is obtained

(13) $$\frac{\partial g(x,y)}{\partial x}=\left\{\begin{array}{cc} -\nu y & 0\leq x\leq \theta \\ -\mu -\nu y & x>\theta \;, \end{array}\right.$$

Therefore, because the parameters ν and μ are positive scalars, for any given $y\in R^{+}$, ∂g/∂x is negative; hence, *g* satisfies point (ii) of Assumption 1.

In the same way, for any given $x\in R^{+}$, ∂g(x,y)/∂y=−νx, which is always negative. This guarantees that *g* satisfies point (iii) of Assumption 1.

**Figure 6:**
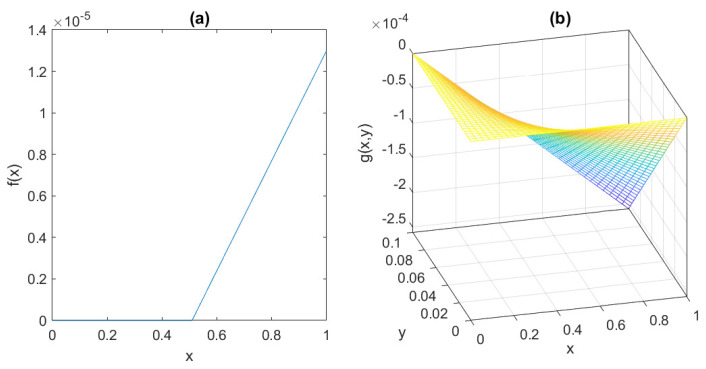
The behaviour of (**a**) *f* and (**b**) *g* in Section 2.4.

The behaviour of the functions *f* and *g* is depicted in Figure 6, assuming the parameter values suggested by [7]:

- γ = 0.095 mU/mgh: insulin production rate of the pancreas;
- ϕ = 0.51 mg/mL: insulin production threshold;
- θ = 2.5 mg/mL: threshold for the discharge of glucose by the kidneys;
- ν = 9.3 mU/mlh: insulin-dependent rate of glucose usage by the tissues;
- μ = 0.48 h${}^{-1}$: glucose excretion rate;
- α = 0.51 h${}^{-1}$: insulin destruction rate of the pancreas;
- β = 0.16 h${}^{-1}$: tissue usage rate of glucose.

Following the review [32], a more realistic and sophisticated model can be obtained, by eliminating the derivative discontinuities in (7) and (10c), replacing the linear fluxes f(·) and $\Phi_{3}\left(\cdot \right)$ by Hill-type functions [47] (see also Section 2.3) in the form

(14a) $$\begin{array}{cc} \hat{f}\left(x\right) & =f_{x}\frac{x^{m_{x}}}{k^{m_{x}}+x^{m_{x}}} \end{array}$$

(14b) $$\begin{array}{cc} {\hat{\Phi }}_{3}\left(x\right) & =-\Phi_{x}\frac{x^{n_{x}}}{h^{n_{x}}+x^{n_{x}}}\;, \end{array}$$

where $f_{x}$, $\Phi_{x}$, *k*, *h*, $m_{x}$, and $n_{x}$ are free parameters to be chosen to approximate the behaviour of *f* and $\Phi_{3}$ given in (7) and (10c), respectively. Note that, independently on the parameter setting, it is guaranteed that $\hat{f}\left(0\right)=0$, $\hat{f}\left(\infty \right)=f_{x}$, ${\hat{\Phi }}_{3}\left(0\right)=0$, and ${\hat{\Phi }}_{3}\left(\infty \right)=-\Phi_{x}$.

**Figure 7:**
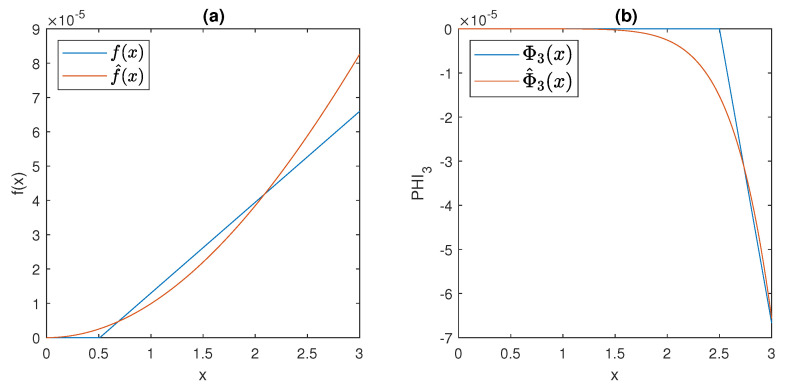
The behaviour of (**a**) activation functions f(x) compared to $\hat{f}\left(x\right)$ and of (**b**) inhibition functions $\Phi_{3}\left(x\right)$ compared to ${\hat{\Phi }}_{3}\left(x\right)$ in Section 2.3.

The plots of $\hat{f}$ and ${\hat{\Phi }}_{3}$, for $f_{x}=10^{-3}$, $\Phi_{x}=1$, k=h=10, $m_{x}=2$, and $n_{x}=8$, are shown in Figure 7, together with the graphs of *f* and $\Phi_{3}$; note that the approximation obtained by the Hill functions is very good in the region of interest, and at the same time, the (rather unrealistic) thresholds are eliminated from the model.

It is readily seen, from the behaviour showed in Figure 7, that, even when the Hill functions are exploited, models (6)–(11) still satisfy Assumption 1.

### 2.5. A Third Paradigmatic Example: The Muscle Stretch Reflex

**Figure 8:**
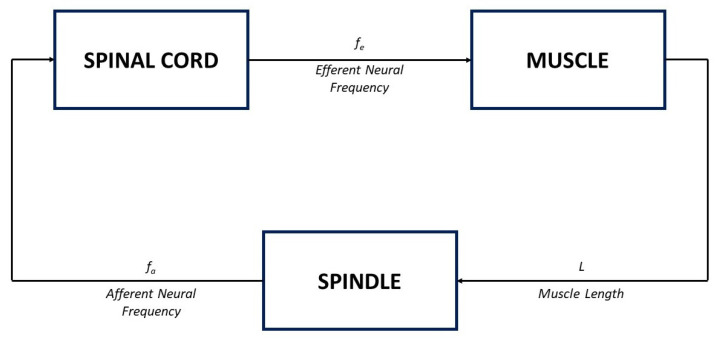
Block diagram of the muscle stretch reflex model in Figure 9.

Here, the model of the muscle stretch reflex, shown in Figure 8, is considered.

**Figure 9:**
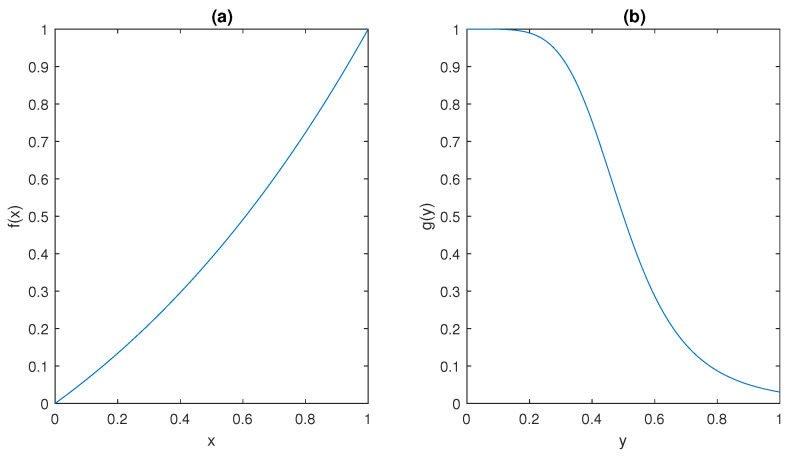
The behaviour of (**a**) *f* and (**b**) *g* (normalised values) in Section 2.5.

According to [54], when the muscle length varies following a tap-induced stretch, neural impulses at a frequency $f_{a}$, which encode information about the magnitude of the stretch, are sent along afferent nerve fibres to the spinal cord (see the spindle block in Figure 8); an increase in *L* corresponds to an increase in the afferent frequency $f_{a}$.

The input–output model of the spindle block can be described by a simple first-order action in the form [7]

(15) $$\dot{f_{a}}=-\gamma f_{a}+0.6L\gamma e^{0.5L}\;.$$

From (15), at steady state ($\dot{f_{a}}=0$), the following equation is obtained:

(16) $$f_{a}=0.6Le^{0.5L}\;,$$

which is consistent with [7]; in (15), the parameter γ captures both the time needed to reach the steady-state value of $f_{a}$ and the transfer delay from the muscle to the spinal cord.

Because each afferent nerve is synaptically connected with one motor neuron in the spinal cord, the motor neurons get activated and, in turn, send efferent neural impulses, at a frequency $f_{e}$, back to the muscle (see the spinal cord block in Figure 8). According to [7], the dynamic of the spinal cord can be neglected, by considering a simple proportional relationship between the input and the output of the corresponding block, in the form [7]

(17) $$f_{e}=kf_{a}\;.$$

Finally, an increase in the efferent neural frequency produces a contraction of the muscle, which translates into a shortening of the muscle length *L* (see the muscle block in Figure 8). The dynamics of the muscle block can be again described by a first-order system, in the form

(18) $$\dot{L}=-\beta L+\frac{0.5^{5}\beta L_{0}}{0.5^{5}+f_{e}^{5}}\;.$$

By letting the derivative be zero, the steady state is obtained; in this case, the input–output relationship between $f_{e}$ and *L*, in agreement with [7], is given by the Hill-type function [47]

(19) $$L=\frac{0.5^{5}L_{0}}{0.5^{5}+f_{e}^{5}}\;,$$

where the parameter β takes into account both the time needed to reach the steady-state value of $f_{e}$ and the transfer delay from the spinal cord back to the muscle.

Without loss of generality, all the variables involved have been scaled to their corresponding maximum values, so that they will range in the interval (0,1); therefore, it is assumed that $L_{0}=k=1$, and the final model, given by the coupling between (15), (17), and (18), takes the form

(20a) $$\begin{array}{cc} \dot{f_{e}} & =-\gamma f_{e}+0.6L\gamma e^{0.5L} \end{array}$$

(20b) $$\begin{array}{cc} \dot{L} & =-\beta L+\frac{0.5^{5}\beta }{0.5^{5}+f_{e}^{5}}\;. \end{array}$$

It is readily seen that, in (20), L=x plays the role of the activation species (i.e., the variation in *L* triggers the reflex arc), while $f_{e}=y$ is the inhibition variable (i.e., the variation in $f_{e}$ brings *L* back close to its starting value); with this notation, the model (20) can be written in the standard form (1)

(21a) $$\begin{array}{cc} \dot{y} & =-\gamma y+0.6x\gamma e^{0.5x}=-\gamma y+f\left(x\right) \end{array}$$

(21b) $$\begin{array}{cc} \dot{x} & =-\beta x+\frac{0.5^{5}\beta }{0.5^{5}+y^{5}}=-\beta x+g\left(y\right)\;, \end{array}$$

where

(22a) $$\begin{array}{cc} f(x) & :=0.6x\gamma e^{0.5x} \end{array}$$

(22b) $$\begin{array}{cc} g(y) & :=\frac{0.5^{5}\beta }{0.5^{5}+y^{5}}\;. \end{array}$$

Note that, in this case, *f* is dependent only on the species *x*, and it is readily seen to satisfy point (ii) in Assumption 1, because it is obtained by the product of two increasing functions and γ is a positive scalar.

On the other hand, *g* is dependent only on the species *y*, and it displays the classical decreasing behaviour of Hill functions; therefore, it satisfies point (iii) in Assumption 1.

### 2.6. Study of the Equilibrium Points

In the present section, the conditions of the existence of feasible equilibrium points for model (1) will be investigated; furthermore, once the existence of at least one equilibrium point is determined, it will be established whether it is exponentially stable.

In order to state the next theorem, it is useful to introduce some definitions concerning the equilibrium points of nonlinear dynamical systems in the form

(23) $$\dot{z}\left(t\right)=h\left(z(t),u(t)\right)\;,$$

where $z\left(t\right)\in R^{n}$ is the system state and $u\left(t\right)\in R^{m}$ is the input.

First, given any $z_{0}\in R^{n}$, the solution of Equation (23), starting from $z_{0}$ at t=0, under the constant input $u\in R^{m}$, is denoted by $z(\cdot ,z_{0},u)$.

Moreover, it is worth recalling that a point $z_{E}\in R^{n}$ is said to be an equilibrium point of system (23), under the constant input $u_{E}\in R^{m}$, if

(24) $$h(z_{E},u_{E})=0\;.$$

**Definition** **1** (Exponentially stable equilibrium point [55]). *Assume that $z_{E}\in R^{n}$ is an equilibrium point of system *(23)* under the constant input $u\left(t\right)=u_{E}\in R^{m}$, that is, $z_{E}$ satisfies *(24)*; then, the equilibrium point $z_{E}$ is said to be exponentially stable with the rate of convergence at least ρ, if there exist positive scalars k, ρ, $d_{z}$, and $d_{u}$, such that for any $z_{0}\in R^{n}$, and $u\in R^{m}$, with*

(25a) $$\begin{array}{cc} ∥z_{0}-z_{E}∥ & \leq d_{z} \end{array}$$

(25b) $$\begin{array}{cc} ∥u-u_{E}∥ & \leq d_{u}\;, \end{array}$$

*the following condition is obtained*

(26) $$∥z\left(t,z_{0},u\right)-z_{E}∥\leq ke^{-\rho t}\;.$$

Given a constant input $u_{E}\in R^{m}$, it is always possible to reduce the stability analysis of the corresponding equilibrium points to a suitable zero-input system, by a change in variable, as follows; let

(27) $$w\left(t\right):=u\left(t\right)-u_{E}\;,$$

so, it is obtained

(28) $$\begin{array}{cc} \dot{z}\left(t\right) & =h\left(z(t),u(t)\right)\\ \; & =h\left(z\left(t\right),w\left(t\right)+u_{E}\right)\\ \; & =:\hat{h}\left(z(t),w(t)\right)\;. \end{array}$$

From (28), it is obtained

(29) $$\begin{array}{cc} \hat{h}\left(z_{E},0\right) & =h(z_{E},u_{E})\\ \; & =0\;,\;\mathrm{from}\;(24)\;; \end{array}$$

therefore, the stability properties of the equilibrium point $z_{E}$ of system (23) under the constant input $u_{E}$ can be equivalently discussed referring to the equilibrium point $z_{E}$ of system (28) under zero input. This fact will be exploited in the proof of the next result.

**Theorem** **1.** *(Exponential stability of the equilibrium points of a physiological control system)*

(*i*) *The set of the equilibrium points of model *(1)* under the constant input ${\left(u_{y}\;u_{x}\right)}^{T}\in R^{2}$ coincides with the set of the solutions of the system of algebraic equations*
  (30a) $$\begin{array}{cc} \alpha y & =u_{y}+f\left(x,y\right) \end{array}$$
  (30b) $$\begin{array}{cc} \beta x & =u_{x}+g\left(x,y\right)\;; \end{array}$$
  *in particular, the equilibrium point is unique if and only if model *(30)* admits a unique solution.*
(*ii*) *Assume that the pair $\left(x_{0},y_{0}\right)$ is an equilibrium point of model *(1)* under the constant inputs ${\left(u_{y}\;u_{x}\right)}^{T}\in R^{2}$, i.e.,*
  (31) $$\alpha y_{0}=u_{y}+f\left(x_{0},y_{0}\right)$$
  (32) $$\beta x_{0}=u_{x}+g\left(x_{0},y_{0}\right)\;;$$
  *then, the point $\left(x_{0},y_{0}\right)$ is an exponentially stable equilibrium point if the following conditions are satisfied:*
  (33a) $$\begin{array}{cc} \alpha +\beta -{\frac{\partial g(x,y)}{\partial x}}_{|\begin{array}{c} x=x_{0}\\ y=y_{0} \end{array}}-{\frac{\partial f(x,y)}{\partial y}}_{|\begin{array}{c} x=x_{0}\\ y=y_{0} \end{array}} & >0\\ \; & \;\left(\alpha -{\frac{\partial f(x,y)}{\partial y}}_{|\begin{array}{c} x=x_{0}\\ y=y_{0} \end{array}}\right)\left(\beta -{\frac{\partial g(x,y)}{\partial x}}_{|\begin{array}{c} x=x_{0}\\ y=y_{0} \end{array}}\right) \end{array}$$
  (33b) $$\begin{array}{cc} -{\frac{\partial f(x,y)}{\partial x}}_{|\begin{array}{c} x=x_{0}\\ y=y_{0} \end{array}}{\frac{\partial g(x,y)}{\partial y}}_{|\begin{array}{c} x=x_{0}\\ y=y_{0} \end{array}} & >0\;. \end{array}$$
(*iii*) *Let*
  (34) $$A:={\left(\begin{array}{cc} -\alpha +\frac{\partial f(x,y)}{\partial y} & \frac{\partial f(x,y)}{\partial x}\\ \frac{\partial g(x,y)}{\partial y} & -\beta +\frac{\partial g(x,y)}{\partial x} \end{array}\right)}_{|\begin{array}{c} x=x_{0}\\ y=y_{0} \end{array}}\;.$$
  *If condition *(33)* is satisfied, assume there exist a positive scalar ρ and a positive definite matrix P, such that the following Linear Matrix Inequality (LMI) [56] is satisfied*
  (35) $$A^{T}P+PA+2\rho P<0\;;$$
  *then, $\left(x_{0},y_{0}\right)$ is an exponentially stable equilibrium point with a rate of convergence at least equal to ρ.*

*Proof:*

**Proof.**  Point (i) immediately follows from the definition of equilibrium point (24).

Concerning point (ii), in order to study the stability of the equilibrium point $\left(x_{0},y_{0}\right)$, it is readily seen that, in this case, the change in input variables (27)–(29) reduces to set the inputs of model (1) to zero, that is, $u_{x}=u_{y}=0$.

Then, the linearised version of model (1), with zero input, around the equilibrium point $\left(x_{0},y_{0}\right)$ is

(36a) $$\begin{array}{cc} \dot{\delta }y\left(t\right) & =-\alpha \delta y\left(t\right)+\frac{\partial f(x,y)}{\partial x}\delta x+\frac{\partial f(x,y)}{\partial y}\delta y \end{array}$$

(36b) $$\begin{array}{cc} \dot{\delta }x\left(t\right) & =-\beta \delta x\left(t\right)+\frac{\partial g(x,y)}{\partial x}\delta x+\frac{\partial g(x,y)}{\partial y}\delta y\;. \end{array}$$

Model (36) can be rewritten in compact form as

(37) $$\left(\begin{array}{c} \dot{\delta }y\left(t\right)\\ \dot{\delta }x\left(t\right) \end{array}\right)=A\left(\begin{array}{c} \delta y(t)\\ \delta x(t) \end{array}\right)\;,$$

where *A* is defined in (34).

The characteristic polynomial associated to matrix *A* is

(38) $$\begin{array}{cc} p(s) & =\det(sI-A)\\ \; & =\det{\left(\left(\begin{array}{cc} s+\alpha -\frac{\partial f(x,y)}{\partial y} & -\frac{\partial f(x,y)}{\partial x}\\ -\frac{\partial g(x,y)}{\partial y} & s+\beta -\frac{\partial g(x,y)}{\partial x} \end{array}\right)\right)}_{|\begin{array}{c} x=x_{0}\\ y=y_{0} \end{array}}\\ \; & \;=s^{2}+\left(\alpha +\beta -{\frac{\partial g(x,y)}{\partial x}}_{|\begin{array}{c} x=x_{0}\\ y=y_{0} \end{array}}-{\frac{\partial f(x,y)}{\partial y}}_{|\begin{array}{c} x=x_{0}\\ y=y_{0} \end{array}}\right)s\\ \; & +\left(\alpha -{\frac{\partial f(x,y)}{\partial y}}_{|\begin{array}{c} x=x_{0}\\ y=y_{0} \end{array}}\right)\left(\beta -{\frac{\partial g(x,y)}{\partial x}}_{|\begin{array}{c} x=x_{0}\\ y=y_{0} \end{array}}\right)\\ \; & -{\frac{\partial f(x,y)}{\partial x}}_{|\begin{array}{c} x=x_{0}\\ y=y_{0} \end{array}}{\frac{\partial g(x,y)}{\partial y}}_{|\begin{array}{c} x=x_{0}\\ y=y_{0} \end{array}}\;. \end{array}$$

Because the characteristic polynomial p(s) is of degree two, a necessary and sufficient condition for having roots with negative real parts is that all the polynomial coefficients are positive; this, in turn, is guaranteed by the hypothesis (33) of the theorem.

Therefore, the linearised model (36) is exponentially stable; according to ([55], Theorem 4.13), this guarantees exponential stability of model (1) under zero input, which in turn implies the exponential stability of the equilibrium point $\left(x_{0},y_{0}\right)$ for the same model under the constant input ${\left(u_{y}\;u_{x}\right)}^{T}$. From this fact, the proof of point ii) follows.

Concerning point (iii), define $\delta z={\left(\begin{array}{cc} \delta y & \delta x \end{array}\right)}^{T}$, the state of the linearised model (36), i.e.,

(39) $$\dot{\delta }z\left(t\right)=A\delta z\left(t\right)\;.$$

Then, condition (50) guarantees that, along the trajectories of model (39), the following condition is held:

(40) $$z^{T}\left(t\right)\left(A^{T}P+PA\right)z\left(t\right)<-2\rho z^{T}\left(t\right)Pz\left(t\right)\;.$$

In this case, the scalar function $V\left(z\right):=z^{T}Pz$ turns out to be a Lyapunov function for the nonlinear model (1), and condition (40) can be rewritten

(41) $$\dot{V}\left(z(t)\right)<-2\rho V\left(z(t)\right)\;,$$

from which it follows that

(42) $$\frac{\dot{V}\left(z(t)\right)}{V\left(z(t)\right)}<-2\rho \;,$$

along model (39) trajectories.

Integrating both sides of (42) between 0 and *t*, it is obtained

(43) $$\begin{array}{cc} \int_{0}^{t}\frac{\dot{V}\left(z(\sigma )\right)}{V\left(z(\sigma )\right)}d\sigma & =\ln\left(\frac{V\left(z(t)\right)}{V\left(z(0)\right)}\right)\\ \; & <-2\rho \;. \end{array}$$

By elevating to *e* both members of (43), the following equation is obtained:

(44) $$V\left(z(t)\right)<V\left(z(0)\right)e^{-2\rho t}\;.$$

Denoted by $\lambda_{min}\left(P\right)$ and $\lambda_{max}\left(P\right)$ the minimum and maximum eigenvalues of the positive definite matrix *P*, and recalling that $V\left(z\right)=z^{T}Pz$, the following chain of inequalities is achieved:

(45) $$\begin{array}{cc} \lambda_{min}\left(P\right){∥z\left(t\right)∥}^{2} & \leq V(z(t))\\ \; & <V\left(z(0)\right)e^{-2\rho t}\;\mathrm{from}\;(44)\\ \; & \leq \lambda_{max}\left(P\right){∥z\left(0\right)∥}^{2}e^{-2\rho t}\;. \end{array}$$

From (45), it follows that

(46) $$∥z\left(t\right)∥\leq {\left[\frac{\lambda_{max}\left(P\right)}{\lambda_{min}\left(P\right)}\right]}^{1/2}∥z\left(0\right)∥e^{-\rho t}\;.$$

From (46), it is possible to conclude, according to Definition 1, that the rate of convergence around $\left(x_{0},y_{0}\right)$ is at least ρ. □

A pair of corollaries of Theorem 1 can be derived, when further assumptions on the functions *f* and *g* hold.

**Corollary** **1.** *(Exponential stability of the equilibrium points when f is not dependent on y)*

*Assume that the activation function f in model *(1)* does not depend on y; then,*

(*i*) *The set of the equilibrium points of model *(1)* under the constant inputs ${\left(u_{y}\;u_{x}\right)}^{T}\in R^{2}$ coincides with the set of the solutions of the system of equations*
  (47a) $$\begin{array}{cc} \alpha y & =u_{y}+f\left(x\right) \end{array}$$
  (47b) $$\begin{array}{cc} \beta x & =u_{x}+g\left(x,y\right)\;; \end{array}$$
  *in particular, the equilibrium point is unique if and only if model *(47)* admits a unique solution.*
(*ii*) *Assume that the pair $\left(x_{0},y_{0}\right)$ is an equilibrium point of model *(1)* under the constant inputs ${\left(u_{y}\;u_{x}\right)}^{T}\in R^{2}$, i.e.,*
  (48a) $$\begin{array}{cc} \alpha y_{0} & =u_{y}+f\left(x_{0}\right) \end{array}$$
  (48b) $$\begin{array}{cc} \beta x_{0} & =u_{x}+g\left(x_{0},y_{0}\right)\;; \end{array}$$
  *then, the point $\left(x_{0},y_{0}\right)$ is an exponentially stable equilibrium point.*
(*iii*) *Let*
  (49) $$A_{x}:={\left(\begin{array}{cc} -\alpha & \frac{df(x)}{dx}\\ \frac{\partial g(x,y)}{\partial y} & -\beta +\frac{\partial g(x,y)}{\partial x} \end{array}\right)}_{|\begin{array}{c} x=x_{0}\\ y=y_{0} \end{array}}\;;$$
  *in this case, assume there exist a positive scalar ρ and a positive definite matrix P, such that the following LMI is satisfied*
  (50) $$A_{x}^{T}P+PA_{x}+2\rho P<0\;;$$
  *then, $\left(x_{0},y_{0}\right)$ is an exponentially stable equilibrium point with a rate of convergence of at least ρ.*

**Proof.**  Point (i) is obvious.

Concerning point (ii), in this case, the application of Theorem 1 guarantees that the equilibrium point $\left(x_{0},y_{0}\right)$ is exponentially stable if the following conditions are both satisfied:

(51a) $$\begin{array}{cc} \alpha +\beta -{\frac{\partial g(x,y)}{\partial x}}_{|\begin{array}{c} x=x_{0}\\ y=y_{0} \end{array}}\; & >0\\ \; & \;\alpha \left(\beta -{\frac{\partial g(x,y)}{\partial x}}_{|\begin{array}{c} x=x_{0}\\ y=y_{0} \end{array}}\right) \end{array}$$

(51b) $$\begin{array}{cc} -{\frac{\partial f(x,y)}{\partial x}}_{|\begin{array}{c} x=x_{0}\\ y=y_{0} \end{array}}{\frac{\partial g(x,y)}{\partial y}}_{|\begin{array}{c} x=x_{0}\\ y=y_{0} \end{array}} & >0\;. \end{array}$$

Because both α and β are positive scalars and ∂g/∂x is non-positive due to condition (ii) in Assumption 1, condition (51a) is always satisfied.

In the same way, because in condition (51b) ∂g/∂x and ∂g/∂y are non-positive and ∂f/∂x is non-negative, due to conditions (ii) and (iii) in Assumption 1, condition (51b) is always satisfied.

From these last considerations, the proof of (ii) follows.

Finally, the proof of (iii) follows the same lines as the proof of (iii) in Theorem 1. □

The next result follows immediately from Theorem 1 and Corollary 1.

**Corollary** **2.** *(Exponential stability of the equilibrium points when f is independent on y and g is independent on x.)*

*Assume that the activation function f in model* (1) *does not depend on y and that the inhibition function g is not dependent on x; then,*

(*i*) *The set of the equilibrium points of model *(1)* under the constant inputs ${\left(u_{y}\;u_{x}\right)}^{T}\in R^{2}$ coincides with the set of the solutions of the system of equations*
  (52a) $$\begin{array}{cc} \alpha y & =u_{y}+f\left(x\right) \end{array}$$
  (52b) $$\begin{array}{cc} \beta x & =u_{x}+g\left(y\right)\;. \end{array}$$
  *Moreover, if g is invertible, the equilibrium points coincide with the intersections of the graphs of the functions*
  (53a) $$\begin{array}{cc} y & =\frac{1}{\alpha }u_{y}+\frac{1}{\alpha }f\left(x\right) \end{array}$$
  (53b) $$\begin{array}{cc} y & =g^{-1}\left(\beta x-u_{x}\right)\;; \end{array}$$
  *in particular, the equilibrium point is unique if and only if the plot of the functions in *(53)* has only one intersection.*
(*ii*) *Assume that the pair $\left(x_{0},y_{0}\right)$ is an equilibrium point of model *(1)* under the constant inputs ${\left(u_{y}\;u_{x}\right)}^{T}\in R^{2}$, i.e.,*
  (54) $$\begin{array}{cc} \alpha y_{0} & =u_{y}+f\left(x_{0}\right) \end{array}$$
  (55) $$\begin{array}{cc} \beta x_{0} & =u_{x}+g\left(y_{0}\right)\;; \end{array}$$
  *then, the point $\left(x_{0},y_{0}\right)$ is an exponentially stable equilibrium point.*
(*iii*) *Let*
  (56) $$A_{xy}:={\left(\begin{array}{cc} -\alpha & \frac{df(x)}{dx}\\ \frac{dg(y)}{\partial y} & -\beta \end{array}\right)}_{|\begin{array}{c} x=x_{0}\\ y=y_{0} \end{array}}\;;$$
  *in this case, assume there exist a positive scalar ρ and a positive definite matrix P, such that the following LMI is satisfied*
  (57) $$A_{xy}^{T}P+PA_{xy}+2\rho P<0\;;$$
  *then, $\left(x_{0},y_{0}\right)$ is an exponentially stable equilibrium point with the rate of convergence at least ρ.*

In general, the exponential stability of an equilibrium point $z_{E}$ of a given nonlinear system is a local property, i.e., it only holds in a neighbourhood of $z_{E}$ (see (25a)); in some situations, it can be important to give an estimate of the domain of attraction of the equilibrium point (see [55], Chapter 4), which is defined as the connected set of initial conditions surrounding $z_{E}$, such that each trajectory, starting from a point in this set, eventually collapses to zero.

To exactly compute the domain of attraction is often a difficult or even impossible task. In many real situations, it is acceptable to have an estimate of the domain; this is possible, for instance, in the case of bilinear [57], quadratic [58], or even polynomial [59] nonlinear systems. Anyway, this issue is beyond the scope of this paper and will be dealt with in future works.

## 3. Results

In this section, the examples considered in Section 2.3 and Section 2.4 will be resumed, in order to apply the theory developed in Section 2.6.

### 3.1. Analysis of the Homeostatic Equilibrium in the Biomolecular Circuit

The model dynamics are described by the ODEs (2) and (3). Observing that, in this case, *f* is not dependent on *x* and *g* is not dependent on *y*, the properties of the equilibrium points can be studied, exploiting Corollary 2.

Taking into account that α=β=1, the inverse of the inhibition function is given by

(58) $$g^{-1}\left(\zeta \right)={\left(\frac{0.4^{4}\left(1-\zeta \right)}{\zeta }\right)}^{1/4}\;,$$

and in view of point (i) of Corollary 2, the equilibrium point is given by the intersection of the curves

(59a) $$\begin{array}{cc} y & =u_{y}+\frac{x^{4}}{0.6^{4}+x^{4}} \end{array}$$

(59b) $$\begin{array}{cc} y & ={\left(\frac{0.4^{4}\left(1-\beta x+u_{x}\right)}{\beta x-u_{x}}\right)}^{1/4}\;. \end{array}$$

**Figure 10:**
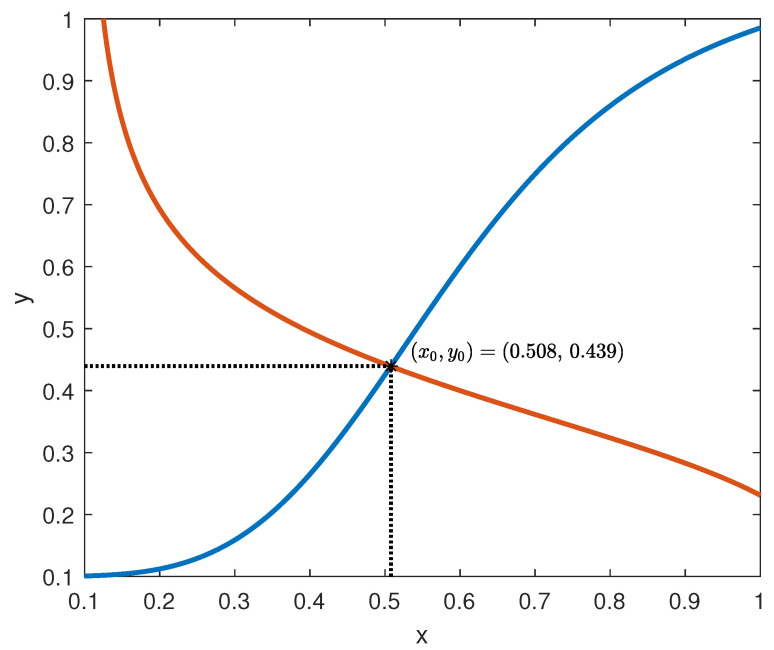
Intersection of the functions in (59).

The intersections of the graphs of the functions in (59), assuming $u_{x}=u_{y}=0.1$ according to [45], are depicted in Figure 10.

From the analysis of Figure 10, it is readily seen that the unique equilibrium point of model (2) is $\left(x_{0},y_{0}\right)=\left(0.508,0.439\right)$, which, according to point (ii) in Corollary 2, is exponentially stable.

In order to evaluate the rate of convergence to the equilibrium, the matrix $A_{xy}$ in (56) has to be computed

(60) $$A_{xy}=\left(\begin{array}{cc} -1.0000 & 1.7655\\ -2.1970 & -1.0000 \end{array}\right)\;,$$

and, to maximise the estimate of the rate of convergence, the following generalised eigenvalue problem (see [56], Ch. 5) has to be solved:

**Problem** **1.** $$\begin{array}{cc} \max\rho \\ \; & s.t.\\ \rho & >0\\ P & >0\\ A_{xy}^{T}P+PA_{xy}+2\rho P & <0\;. \end{array}$$

With the aid of the Matlab LMI toolbox [60], Problem 1 is solved with the optimal solution given by

(61) $$\rho^{★}=0.99\;,\;P^{★}=\left(\begin{array}{cc} 56.1100 & -0.0278\\ -0.0278 & 45.0900 \end{array}\right)\;.$$

**Figure 11:**
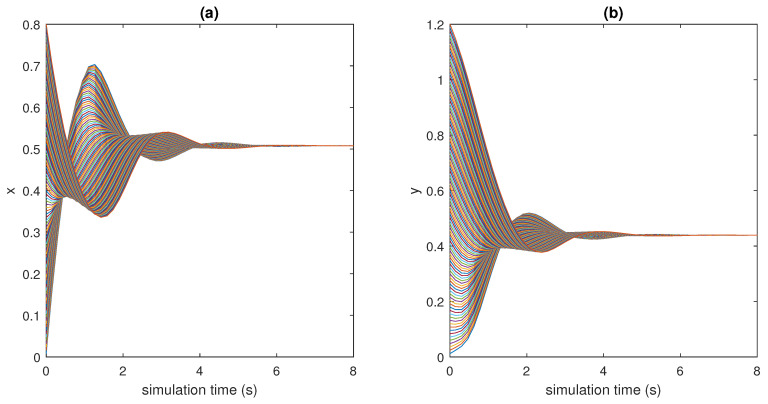
State variables of models (2) and (3) as functions of the simulation time: (**a**) *x* vs. time; (**b**) *y* vs. time.

**Figure 12:**
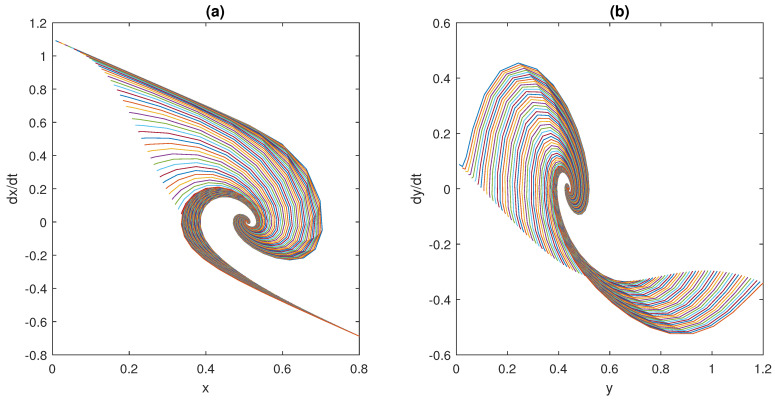
Phase plots for the state variables of models (2) and (3): (**a**) $\frac{dx}{dt}$ vs. *x*; (**b**) $\frac{dy}{dt}$ vs. *y*.

**Figure 13:**
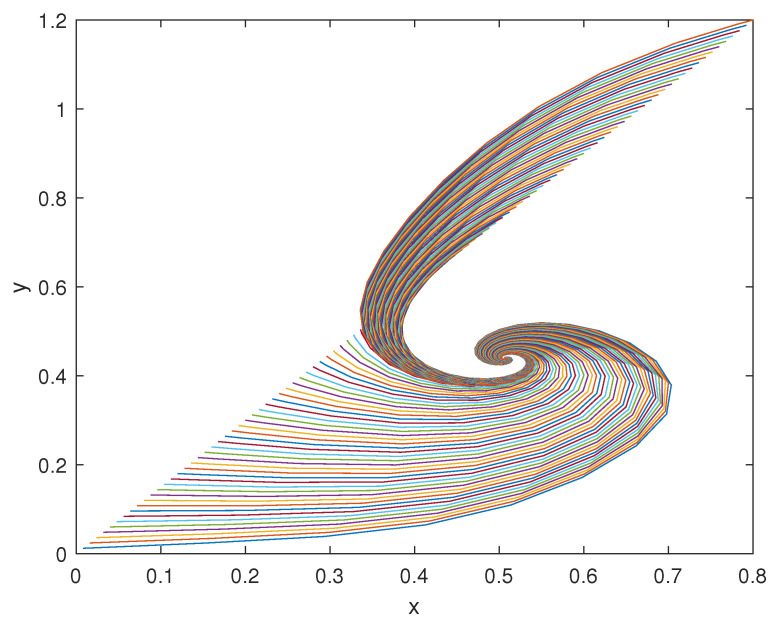
Phase-state plot of models (2) and (3): *y* vs. *x*.

The trajectories of model (2) around the equilibrium point $\left(x_{0},y_{0}\right)$, with the expected rate of convergence, are shown in Figure 11, Figure 12 and Figure 13. In particular, 100 different trajectories are simulated by changing the initial conditions of the model in a range between 0 and 1 for both *x* and *y*. As expected from the theoretical results, the trajectories converge exponentially, with the expected rate, to the equilibrium point $\left(x_{0},y_{0}\right)=\left(0.508,0.439\right)$.

**Remark** **4.** *The current example is useful to understand the rationale for which Assumptions 1-(ii) and 1-(iii) are introduced (see also Remark 1). The fact that f is non-decreasing and g is not increasing ensures that, around the equilibrium point, two opposite behaviours are exhibited (see Figure 10) that tend to bring back the system to the equilibrium, whenever a perturbation occurs.*

### 3.2. Analysis of the Homeostatic Equilibrium in Blood Glucose Regulation

Both the activation (see (6)) and inhibition (see (11)) dynamics have been previously described, and because, as observed in Section 2.4, the activation function showed to be dependent only on the *x* species, it is possible to apply Corollary 1. The parameter values suggested by [7] and shown in Section 2.4 are assumed.

By solving numerically (48), the following equilibrium point for the system under study, $\left(x_{0},y_{0}\right)=\left(0.810,0.056\right)$, is obtained, with $x_{0}$ given in mg/mL and $y_{0}$ in mU/mL; such an equilibrium point, in view of point (i) of Corollary 1, is given by the intersection of the curves

(62a) $$\begin{array}{cc} y & =\left\{\begin{array}{cc} 0 & 0\leq x\leq \phi \\ \frac{\gamma (x-\phi )}{\alpha } & x>\phi \;, \end{array}\right. \end{array}$$

(62b) $$\begin{array}{cc} y & =\left\{\begin{array}{cc} \frac{\left(\beta x-u_{x}\right)}{-\nu x} & 0\leq x\leq \theta \\ \frac{\left(\beta x-u_{x}\right)+\mu \left(x-\theta \right)}{-\nu x} & x>\theta \;. \end{array}\right. \end{array}$$

**Figure 14:**
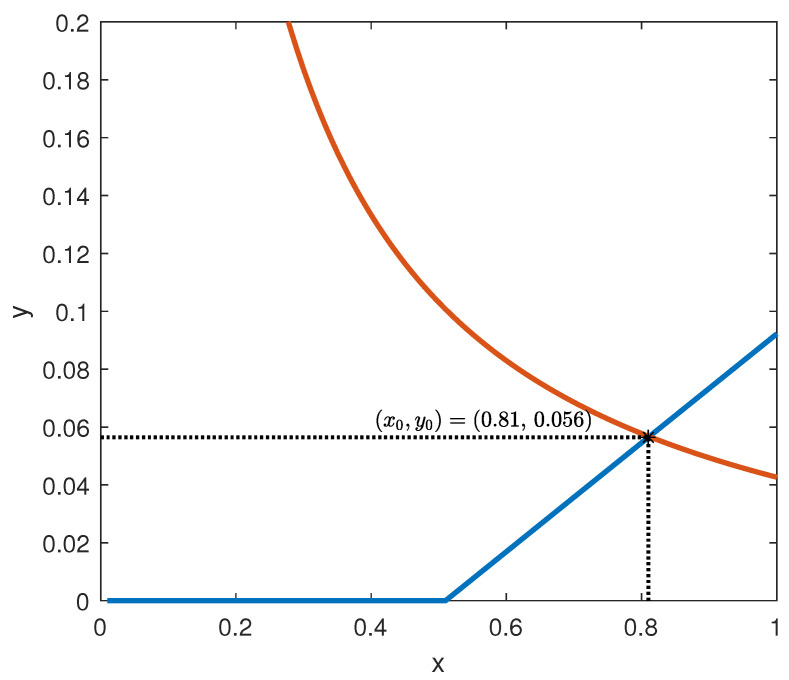
Intersection of the functions in (62).

The intersection of the graphs of the functions in (62), assuming $u_{x}=0.56$, expressed in mg/mlh (glucose produced by the liver by glycogenolysis), according to [7], is depicted in Figure 14.

The application of point (ii) in Corollary 1 ensures the exponential stability of the equilibrium point.

In order to evaluate the rate of convergence to the equilibrium, the matrix $A_{x}$ in (49) has to be computed

(63) $$A_{x}=10^{-4}\left(\begin{array}{cc} -1.4074 & 0.2648\\ -0.0021 & -1.9104 \end{array}\right)\;,$$

and, as in the previous example, to maximise the estimate of the rate of convergence, the generalised eigenvalue Problem 1 (with $A_{x}$ replacing $A_{xy}$) is solved.

In this case, the optimal solution is given by

(64) $$\rho^{★}=0.3564\;h^{-1}\;,\;P^{★}=\left(\begin{array}{cc} 6.7487 & -0.0096\\ -0.00960 & 0.0005 \end{array}\right)\;.$$

**Figure 15:**
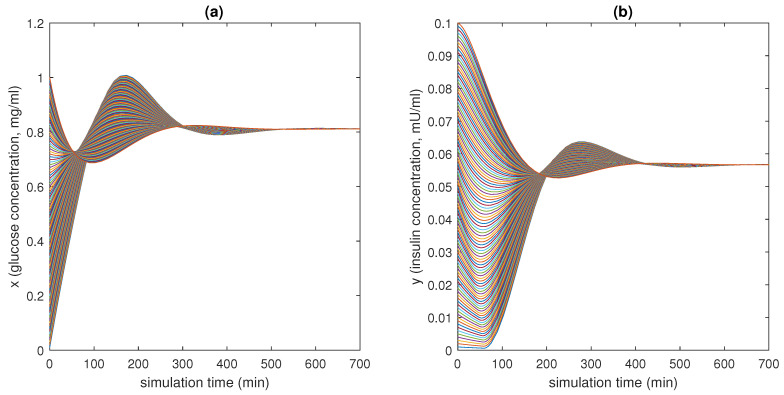
State variables of models (6)–(11) as functions of the simulation time: (**a**) concentration of blood glucose, *x* (in mg/mL), vs. time; (**b**) concentration of insulin, *y* (in mU/mL), vs. time.

**Figure 16:**
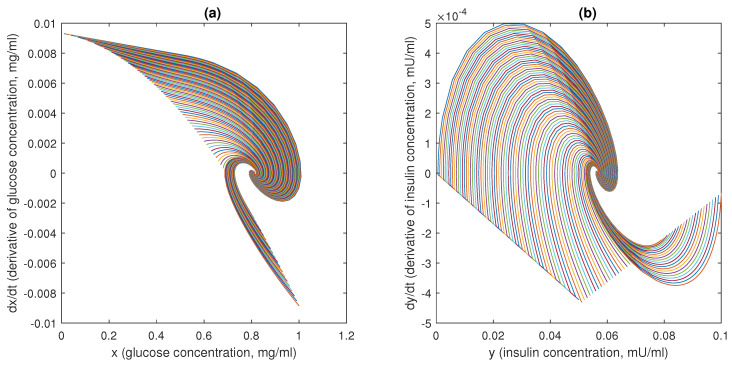
Phase plots for the state variables of models (6)–(11): (**a**) variation in plasma glucose concentration, $\frac{dx}{dt}$, vs. *x* (in mg/mL); (**b**) variation in insulin concentration, $\frac{dy}{dt}$, vs. *y* (in mU/mL). Reproduced with permission from [41].

**Figure 17:**
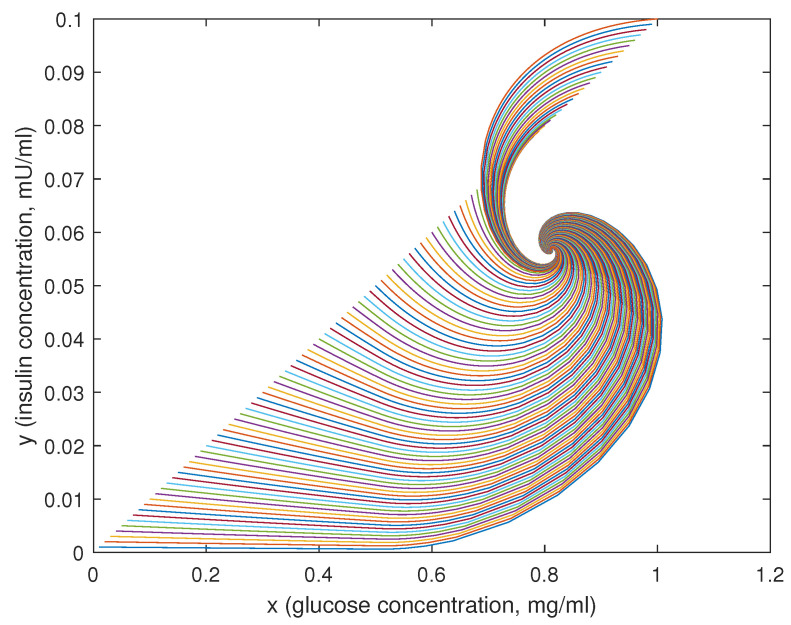
Phase-state plot of models (6)–(11): concentration of insulin, *y* (in mU/mL), vs. concentration of blood glucose, *x* (in mg/mL).

In Figure 15, Figure 16 and Figure 17, 100 different trajectories around the equilibrium point $\left(x_{0},y_{0}\right)$ are depicted, starting from different initial conditions (from 0 to 1 mg/mL for the *x* species and from 0 to 0.1 mU/mL for the *y* species). According to the theoretical results, all the simulated trajectories converge exponentially, with the expected rate, to the equilibrium point $\left(x_{0},y_{0}\right)=\left(0.810,0.056\right)$.

For the sake of completeness, it is interesting to repeat the analysis by replacing the linear fluxes f(·) and $\Phi_{3}\left(\cdot \right)$ given in (7) and (10c) by the Hill-type functions (14) [47].

In this case, the only admissible equilibrium point, given by the intersection of the curves

(65a) $$\begin{array}{cc} y & =\frac{10^{-3}}{\alpha }\;\frac{x^{2}}{10^{2}+x^{2}} \end{array}$$

(65b) $$\begin{array}{cc} y & =\frac{1}{\nu x}\left[-\beta x+u_{x}+\mu \left(x-\theta \right)-\frac{x^{8}}{10^{8}+x^{8}}\right]\;, \end{array}$$

is $\left({\hat{x}}_{0},{\hat{y}}_{0}\right)=\left(0.86,0.052\right)$, which turns out to be very close to the previous one, namely, $\left(x_{0},y_{0}\right)=\left(0.81,0.056\right)$.

By computing the new dynamical matrix ${\hat{A}}_{x}$ in (49)

(66) $${\hat{A}}_{x}=10^{-4}\left(\begin{array}{cc} -1.4074 & 0.1695\\ 0.0000 & -0.4574 \end{array}\right)\;,$$

and by solving the generalised eigenvalue Problem 1, the new estimate of the rate of convergence is

$${\hat{\rho }}^{★}=0.3239\;h^{-1}\;,$$

which is comparable to the one previously obtained in (64).

These latter results show that the simpler model given from (6)–(10) captures all the fundamental dynamics of the blood glucose regulation model, while, in spite of an increasing computational burden, the adoption of the more sophisticated model, where the Hill functions (14) are exploited, does not add meaningful knowledge to the investigation.

### 3.3. Analysis of the Homeostatic Equilibrium in the Muscle Stretch Reflex

The model dynamics are described by the ODEs (21). Similar to the biomolecular circuit model (first paradigmatic example), in this case, *f* and *g* are not dependent on *x* and *y*, respectively. Therefore, the results of Corollary 2 will be exploited.

By assuming the following values for γ and β (from [61])

$$\gamma =\frac{1}{7}\;10^{3}\;s^{-1}\;,\;\;\beta =\frac{1}{10}\;10^{3}\;s^{-1}\;,$$

in view of point (i) of Corollary 2, the equilibrium point is given by the intersection of the curves

(67a) $$\begin{array}{cc} y & =0.6xe^{0.5x} \end{array}$$

(67b) $$\begin{array}{cc} y & ={\left(\frac{0.5^{5}-0.5^{5}x}{x}\right)}^{1/5}\;. \end{array}$$

**Figure 18:**
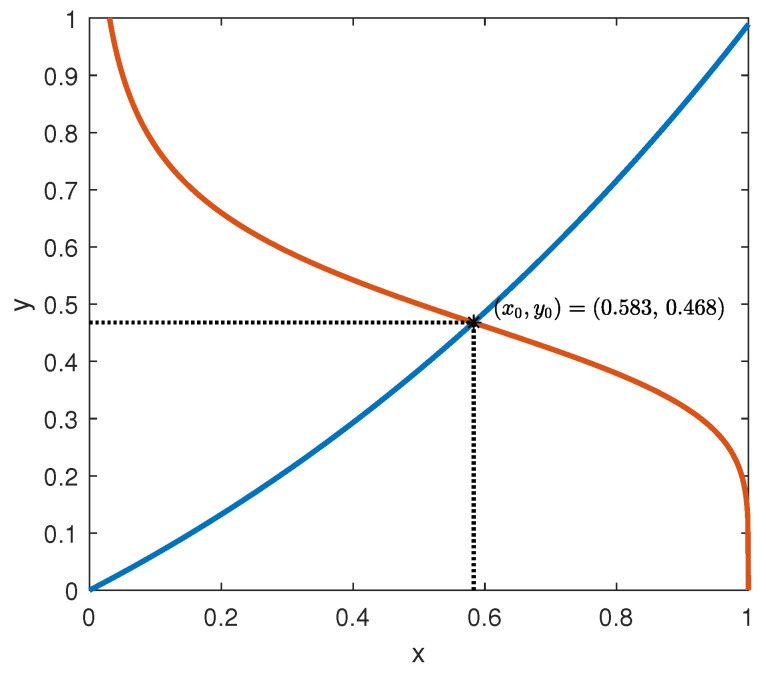
Intersection of the functions in (67).

The intersection of the graphs of the functions in (67) is depicted in Figure 18. It is readily seen that the unique equilibrium point of model (21) is $\left(x_{0},y_{0}\right)=\left(0.583,0.468\right)$, which, according to point (ii) in Corollary 2, is exponentially stable.

The rate of convergence to the equilibrium is calculated by computing the matrix $A_{xy}$ in (56)

(68) $$A_{xy}=\left(\begin{array}{cc} -142.86 & 148.16\\ -259.90 & -100.00 \end{array}\right)\;,$$

and by solving the generalised eigenvalue Problem 1, to obtain the estimated rate of convergence

(69) $$\rho^{★}=123.28\;s^{-1}\;,\;P^{★}=10^{-6}\left(\begin{array}{cc} 0.1472 & 0.0000\\ 0.0000 & 0.1472 \end{array}\right)\;.$$

**Figure 19:**
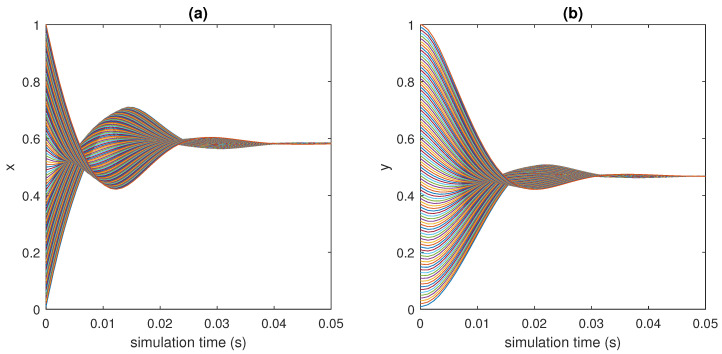
State variables of model (21) as functions of the simulation time: (**a**) *x* vs. time; (**b**) *y* vs. time.

**Figure 20:**
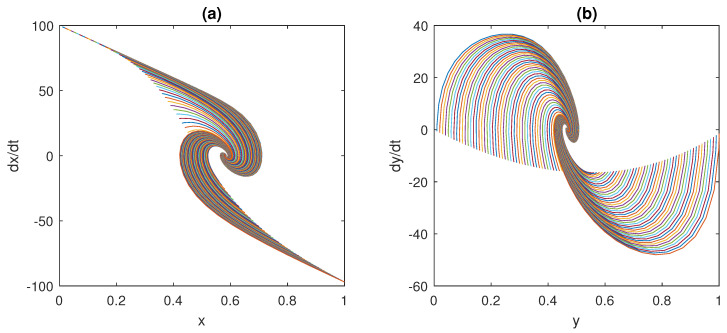
Phase plots for the state variables of model (21): (**a**) $\frac{dx}{dt}$ vs. *x*; (**b**) $\frac{dy}{dt}$ vs. *y*.

**Figure 21:**
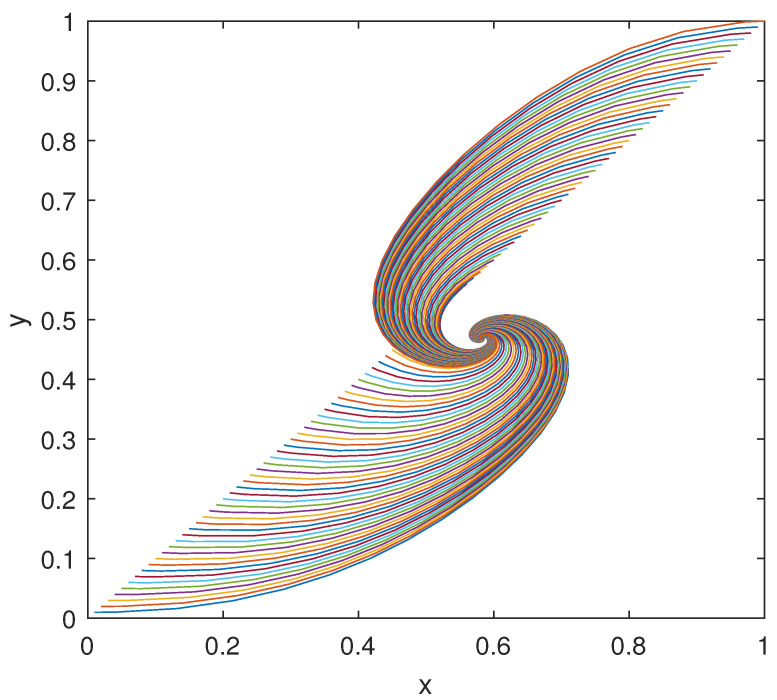
Phase-state plot of model (21): *y* vs. *x*.

The trajectories of model (21) around the equilibrium point $\left(x_{0},y_{0}\right)$, with the expected rate of convergence, are shown in Figure 19, Figure 20 and Figure 21, where 100 trajectories are simulated by changing the initial conditions between 0 and 1 for both *x* and *y*. All the trajectories converge exponentially to the equilibrium point $\left(x_{0},y_{0}\right)=\left(0.583,0.468\right)$ at the estimated rate $\rho^{★}$.

## 4. Discussion

Within the dynamics of physiological processes, the maintenance of the homeostatic balance is obviously of fundamental importance. It is a key process to keep some variables constant on average. Different inputs (or disturbances) cause deviations from the steady state. The degree of deviation can be weak under some circumstances and substantial in others. Sometimes the homeostatic process is not able to recover, and a pathological condition is generated. For this reason, in the literature, many papers that try to explain this mechanism have been published; however, for historical and cultural reasons, most of these articles are clinical and therefore explain how homeostasis is implemented at the cellular level (see, e.g., [35,36,37]). On the other hand, in the last twenty years, there has also been an increasing number of papers that try to describe the mechanism through mathematical models (see, among others, [32,38,39,40,62,63,64]). Nevertheless, these latter papers deal with specific cases and are often very complex. Moreover, some studies, which nominally exploit mathematical models, only introduce a formalism based on block schemes (e.g., see [40]); conversely, the approach provided in this work is precisely what allows the treatment to be generalised.

Indeed, a unique and general nonlinear model of the second order is proposed and it is represented by means of two ODEs, each one containing the following: (i) a consumption term that is linearly dependent on the corresponding state variable (−αy and −βx, respectively), exhibiting a stabilising effect on the system, and (ii) a coupling term that considers the two species interactions through an activation (f(x,y)) and an inhibition (g(x,y)) channel. Through this model, the conditions determining the existence and stability of equilibrium points have been investigated and established. Such conditions proved to be pretty straightforward in their application in the presence of a *y*-independent activation function *f* (Corollary 1), and in the presence of an *x*-independent inhibition function *g* (Corollary 2); in the last case, a simple graphical procedure allows to determine the presence of equilibrium points and their possible uniqueness. Also, a method to estimate the rate of convergence of the state trajectories to the equilibrium has been provided.

To the best of our knowledge, this is the first time that the development of a general formulation for these types of processes is tackled.

In this context, the case studies here considered show that model (1) is able to capture the dynamics of the physiological systems dealt with, namely, the ultra-sensitivity action exhibited by biomolecular circuits, the regulation of glucose concentration in the plasma, and the neuromuscular reflex arc.

Obviously, the consideration above is not sufficient to conclude that through model (1) it will be possible to describe the majority of physiological systems from the nano- to the macro-scale. However, these results make us confident that such a generalisation is possible without employing complex models and avoiding customisation, by differentiating them from the beginning. Finding a unique general model, capable of capturing the dynamics of many physiological mechanisms active in mammals to maintain the homeostatic equilibrium, by changing the choice of some functions, is the long-term goal of this study. This work must be seen as the first encouraging step towards this objective. Encouragement comes also from the values obtained at the equilibrium point, and the rates of convergence of the investigated systems, which are all consistent with those reported in the scientific literature and physiology books. These results represent a validation of the proposed model in three different examples of physiological control systems that are relevant in the bioengineering field. Furthermore, as mentioned in the Introduction, while the computational time required to solve mathematical models grows with the order of the model itself, the framework here proposed can be solved by means of readily available simulation tools (such as MATLAB Simulink) and with low computational efforts (the time to run and solve the models takes a few seconds). This is not infrequent in the modelling of physiological systems and pushes researchers towards the development of simpler, in place of computationally heavy, models. Obviously, this represents an advantage also compared to in vivo or in vitro experiments, which are even more complex and time-consuming.

The authors are confident that model (1) contains much of the ingredients to render quite general the model itself; it is worth noting that the first (second) ODE in (1) contains a negative linear term, namely, −αy (−αx), which accounts for the intrinsic stabilising effect due to the species consumption, plus an activatory (inhibitory) coupling with the second (first) ODE, which looks at the stabilisation of the whole system (see Remarks 1 and 4). These actions, to the best of the authors’ knowledge, are the main functions, which have a role in any physiological negative feedback control scheme.

Anyway, further work will be required in the future, by investigating many more physiological control systems, with the aim of further validating the model (1) and/or adding more complexity, whenever it will be necessary, in order to render the model itself as general as possible. The first issue will be the possible coupling between the inputs, i.e., $u_{x}$ and $u_{y}$, and some (or all) of the state variables, i.e., *x* and *y*, which would translate into possible products between the involved variables, e.g., $u_{y}x$ or $u_{y}y$.

Another important issue will be the study of the domain of attraction of the equilibrium points (see the discussion at the end of Section 2). The estimation of this domain is important in many contexts. For instance, it defines the limit beyond which the system can no longer self-regulate and therefore moves into a pathological condition (e.g., see [65] and the bibliography therein). Moreover, some genetic circuits can exhibit both a mono-stable and bi-stable behaviour [10,66]; in the latter case, they work as a biological switch, where each equilibrium represents one of the two states (on and off); in that case, the stability region of each equilibrium state must be determined with a certain accuracy.

A further important step will be the generalisation of model (1) to the multivariable case, when the variables *x* and *y* are vectors rather than scalars. If, from one point of view, the formalism remains quite unchanged, the vector nature of *x* and *y* complicates the mathematical machinery contained in Theorem 1 and the corollaries. On the other hand, such generalisation will allow us to greatly enlarge the class of physiological systems covered by the proposed approach.

Finally, in addition to the efforts devoted to the generalisation and validation of the proposed model, a systematic review of the literature could be carried out in the future to analyse other different existing models and compare their strengths and weaknesses.

## 5. Conclusions

A general framework for the modelling of physiological control systems, which depends on the interplay of two species (*x* and *y*), is introduced and investigated. For these systems, sufficient conditions determining both the existence and the exponential stability of feasible equilibrium points have been investigated and established.

The proposed approach has been illustrated through three real physiological examples. The first one deals with the ultrasensitivity dynamics observed in many different molecular mechanisms, the second one with the physiological model of blood glucose regulation, and the third one with the neuromuscular reflex arc model. In all cases, an extensive analysis, based on the application of Theorem 1 and its corollaries, has been carried out, showing that the proposed approach well-captures the main mechanisms underlying the physiological control systems dealt with in this work. Indeed, the values at the equilibrium point as well as the time necessary to reach them are consistent with those reported in the scientific literature and physiology books [7,34].

Future work will be devoted to investigating the generalisation of the proposed model to multi-state variables systems, to the development of a methodology to estimate the region of attraction of the equilibrium points, and, overall, to validate, and eventually improve, the proposed model versus further physiological negative feedback control systems, from the nano- to the macro-scale.

## Data Availability

Not applicable.
